# Retraction Note to: LncRNA PVT1 triggers Cyto-protective autophagy and promotes pancreatic ductal adenocarcinoma development via the miR-20a-5p/ULK1 Axis

**DOI:** 10.1186/s12943-022-01678-7

**Published:** 2022-11-11

**Authors:** Fengting Huang, Wenying Chen, Juanfei Peng, Yuanhua Li, Yanyan Zhuang, Zhe Zhu, Chunkui Shao, Wanling Yang, Herui Yao, Shineng Zhang

**Affiliations:** 1 Department of Gastroenterology and Guangdong Provincial Key Laboratory of Malignant Tumor Epigenetics and Gene Regulation, Sun Yat-sen Memorial Hospital, Sun Yat-sen University, No. 107 Yanjiang West Road, Guangzhou, 510120 China; 2 Department of Gastroenterology, Sun Yat-sen Memorial Hospital, Sun Yat-sen University, Guangzhou, 510120 China; 3 Department of Medicine, Division of Regenerative Medicine, University of California, San Diego, School of Medicine, La Jolla, CA 92093 USA; 4 Department of Pathology, The Third Affiliated Hospital, Sun Yat-sen University, Guangzhou, 510630 China; 5 Department of Paediatrics and Adolescent Medicine, Centre for Genomic Sciences, LKS Faculty of Medicine, The University of Hong Kong, Pokfulam, Hong Kong; 6 Department of Medical Oncology, Sun Yat-sen Memorial Hospital, Sun Yat-sen University, No. 107 Yanjiang West Road, Guangzhou, 510120 China

**Retraction note to: Mol Cancer 17, 98 (2018)**

**https://doi.org/10.1186/s12943-018-0845-6**

The Editor in Chief has retracted this article. After publication, concerns were raised in regards to some figures, specifically:

· Fig. 4C: in the Hoechst 33342 row, the LV-PVT1 panel of Capan-2 appears to correspond to the sh-PVT1-1+rapa panel of HPAF-II with a rotation and background brightness difference;
· Fig. 4C: there appears to be a partial overlap between HPAF-II panels for sh-NC and sh-PVT1-1 in all three rows;
· The LV-PVT1+3MA plot in Fig. 4E is unexpectedly similar to the miR20a+PVT1 plot in Fig. S4C.

The authors did not provide a sufficient explanation. Additionally, it appears the authors initially obtained only a provisional “fast-track” ethics approval for the study and applied for full approval at revision stage. This is not in line with journal policy.

Therefore, the Editor in Chief has lost confidence in the integrity of the article's findings. Fengting Huang, Wenying Chen, Juanfei Peng, Yuanhua Li, Wanling Yang, Herui Yao and Shineng Zhang agree to this retraction. Yanyan Zhuang, Zhe Zhu and Chunkui Shao did not respond to correspondence from the Editor about this retraction.

